# Limitations of a proper SFTSV mouse model using human C-type lectin receptors

**DOI:** 10.3389/fmicb.2024.1452739

**Published:** 2024-12-19

**Authors:** You-Min Kim, Hyo-Jin Ro, Jae Hoon Lee, Yaechan Song, Han-Woong Lee, Nam-Hyuk Cho

**Affiliations:** ^1^Department of Biochemistry, College of Life Science and Biotechnology, Yonsei University, Seoul, Republic of Korea; ^2^Department of Biomedical Sciences, Seoul National University College of Medicine, Seoul, Republic of Korea; ^3^Department of Microbiology and Immunology, Seoul National University College of Medicine, Seoul, Republic of Korea; ^4^GEMCRO, Inc., Seoul, Republic of Korea; ^5^Institute of Endemic Diseases, College of Medicine, Seoul National University, Seoul, Republic of Korea; ^6^Seoul National University Bundang Hospital, Seongnam-si, Gyeonggi-do, Republic of Korea

**Keywords:** severe fever thrombocytopenia syndrome virus, DC-SIGN, DC-SIGNR, LSECtin, transgenic mice

## Abstract

Severe fever with thrombocytopenia syndrome virus (SFTSV) is a tick-borne virus with a human mortality rate of up to 30%, posing a significant threat to public health. However, the lack of suitable research models has impeded the development of effective human vaccines. In this study, we engineered transgenic mice (3xTg) using a novel construct that simultaneously expresses three C-type Lectin receptors, identified as critical SFTSV entry receptors. While this construct substantially enhanced viral binding and infection in BJAB cells, the 3xTg mice exhibited only limited SFTSV replication in the lymph nodes and spleen, without significant impacts on morbidity or mortality. These findings highlight that the overexpression of entry receptors alone is insufficient to fully recapitulate human SFTSV infection in mice. Moreover, our results reveal that the introduction of multiple entry receptors does not necessarily translate to enhanced infection efficacy. This underscores the need for further investigation into the interplay between SFTSV entry mechanisms and host factors to develop more robust mouse models. Advancing such models will be crucial for unraveling the pathogenesis of SFTS pathology and improving strategies for its prevention and treatment in humans.

## 1 Introduction

The recurring emergence of viral epidemics, such as coronavirus disease, underscores the substantial threat to human public health and their profound impact on human populations. Recent evaluations by the World Health Organization have emphasized the potential for pandemics originating from arboviruses (Balakrishnan, 2022; Zhang et al., 2023). Among these threats, the severe fever with thrombocytopenia syndrome virus (SFTSV), a member of the Phenuiviridae family (formerly Bunyaviridae), is particularly concerning due to its high mortality rate of 12 to 30% in humans (Sun et al., 2021; Yamada et al., 2018). Since its initial identification in China in 2009, SFTSV has progressively affected a growing number of individuals across East Asia and has expanded to new areas, including the United States of America and Australia (Sun et al., 2021; Zhang et al., 2023; Miao et al., 2020). SFTS is characterized by severe fever, hemorrhage, thrombocytopenia, leukocytopenia, and multiple organ failure, all contributing to its high mortality rate (Li, 2011; Li et al., 2018). Despite its clinical importance, the lack of effective treatments for SFTSV remains ineffective by an incomplete understanding of host factors, particularly the cellular entry receptors (Zhang et al., 2022). Moreover, the absence of suitable SFTSV animal models limits our understanding of its pathogenesis and host immune response, hindering the development of an effective vaccine (Sun et al., 2021; Tani et al., 2016a; Tian et al., 2021).

SFTSV comprises a negative-sense single-stranded RNA (ssRNA) genome segmented into large (L), medium (M), and small (S) segments (Casel et al., 2021; Zhang et al., 2022). The M segment encodes a membrane glycoprotein precursor (Gp), cleaved into glycoprotein N (Gn) and glycoprotein C (Gc) (Casel et al., 2021; Zhang et al., 2022). These glycoproteins form spike-like structures that facilitate viral attachment to host cells and penetration through membrane fusion (Lozach et al., 2011). Accordingly, recent efforts have led to the identification of viral entry receptors that facilitate the binding of glycoproteins to cell surface receptors.

Dendritic cell-specific intercellular adhesion molecule-3-grabbing non-integrin (DC-SIGN), DC-SIGN receptor (DC-SIGNR), and liver sinusoidal endothelial cell lectin (LSECtin) are C-type lectins with a high affinity for high-mannose oligosaccharides (Mason and Tarr, 2015; Guo et al., 2004; Brown et al., 2018). C-type lectins contain a carbohydrate-recognition domain that binds to high-mannose structures present in viral envelope glycoproteins (Mason and Tarr, 2015; Guo et al., 2004). C-type lectins are distinguished in tissue expression, ligand affinities, structure, and function (Mason and Tarr, 2015). C-type lectin, DC-SIGN is a type II transmembrane lectin receptor found on dendritic cells (DCs) that seizes and internalizes antigens (Svajger et al., 2010; Feinberg et al., 2001). DC-SIGN has been identified as a significant endocytic factor in the entry mechanism of SFTSV (Suzuki et al., 2020; Hofmann et al., 2013; Tani et al., 2016b; Tani et al., 2019). DC-SIGNR has 77% homology with DC-SIGN (Pohlmann et al., 2001). Although similar to DC-SIGN, DC-SIGNR can also attach to high-mannose glycans and the same pathogens, and is abundantly expressed in the endothelial cells of the liver and lymph nodes (Guo et al., 2004; Tani et al., 2016b; Tani et al., 2019). Another C-type lectin, LSECtin has been identified as an SFTSV receptor (Tani et al., 2016b; Dominguez-Soto et al., 2007; Zhang et al., 2014; Gramberg et al., 2008; Tani et al., 2019). LSECtin is detected mainly in the sinusoidal endothelial cells of the liver, lymph nodes, macrophages, and DCs (Dominguez-Soto et al., 2007). In addition, LSECtin has function to inhibit T cell activation and proliferation and to reduce the production of cytokines and chemokines in activated T cells such as IFN-γ, IL-2, IL-17, and TNF-α (Wang et al., 2021). Accordingly, the overexpression of these three human C-type lectins could therefore increase susceptibility to SFTSV infection in mice.

While each of the C-type lectins has been identified as an SFTSV entry receptor and used for SFTSV vaccine development, transiently expressing a single SFTSV entry receptor was insufficient to maintain SFTSV infection for more than 8 days and to mediate late symptoms of SFTSV infection to research SFTS therapeutics (Park et al., 2023). In this study, we established a transgenic mouse expressing three human C-type lectins-DC-SIGN, DC-SIGNR, and LSECtin-within an intact immune system to provide an appropriate mouse model for extended SFTSV infection studies. SFTSV transgenic (3xTG) mice successfully express these entry receptors in key organs such as the spleen, kidney, liver, and lungs, rendering them susceptible to SFTSV infection. However, our findings indicate that even the combined expression of these receptors does not fully replicate the human pathological response to SFTSV. This suggests that simply increasing the number of entry receptors does not necessarily enhance the efficacy of infection (Lozach et al., 2004). Alternatively, this limited response may be due to excessive immune protection against viral infections in mice (Schetters et al., 2018; Schaefer et al., 2008), causing rapid eradication of viral pathogens before being effect (Brown et al., 2018). Thus, our findings show that, although entry receptors enable SFTSV infection in mice, controlling pathogen clearance is critical for successfully imitating human SFTS in mice.

## 2 Materials and methods

### 2.1 Transgene cloning, microinjection, and generation of whole-body transgenic mouse

Full-length cDNA expression plasmids for human CD209/DC-SIGN-His, DC-SIGNR/CD299, and CLEC4G (LSECtin) were purchased from Sino Biological (cat. no. HG10200-CH, HG10559-UT, and HG20770-UT). The cDNAs were subjected to polymerase chain reaction (PCR) amplification and cloned into the pCAGGS vector. The plasmid was linearized using *Sal*I and *Bam*HI (NEB, Ipswich, MA, USA) for microinjection into mouse embryos. Microinjections were performed as described previously.

### 2.2 Cell culture and virus

HEK 293T cells were purchased from the American type culture collection (ATCC). All cells were cultured in Dulbecco’s Modified Eagle’s Medium (DMEM; Gibco) supplemented with 10% fetal bovine serum (FBS; Sigma) and 1% penicillin/streptomycin (Gibco) at 37°C in a humidified chamber containing 5% CO2. Vero E6 cells (ATCC CRL-1586) were maintained in DMEM (Welgene) supplemented with 10% (FBS; Gibco) and 1% PS (Gibco). BJAB cells were cultured in Roswell Park Memorial Institute medium (RPMI-1640; Welgene) containing 10% (FBS; Gibco) and 1% PS (Gibco). Wild-type SFTSV strain 2015-JJ01 (NCBI Accession number: MN329148-329140) was isolated from a patient in South Korea. For the *in vitro* infectivity assays, SFTSV containing the green fluorescent protein (GFP) gene, excluding the Ns gene, was used. Recombinant SFTSV was generated using a reverse genetics system as previously described (Hofmann et al., 2013). Viruses used in this study were propagated in Vero E6 cells. The supernatant from the infected cells was harvested 4 days after infection and stored at -80°C after filtering through a 0.45 μm syringe filter. The viral infection titer was determined using a focus-forming assay and presented as focus-forming units (FFU).

### 2.3 Transfection

HEK293T cells at 60–70% confluency were transfected with 1 μg pCAG-DC-SIGN DNA, pCAG-DC-SIGNR DNA, pCAG-LSECtin DNA, or pCAG-DC-SIGN-P2A-DC-SIGNR-T2A-LSECtin DNA (pCAG-3xSFTSVR) 24 h after seeding using 3 μg OmicsFect™ *in vitro* transfection Reagent (Omics Bio, CP2101). Two hours after the transfection, the medium was replaced. BJAB cells were transfected using the Neon Transfection System (Invitrogen), following the manufacturer’s protocol. Briefly, 10^6^ cells were transfected with 10 μg of pCAG-DC-SIGN, pCAG-DC-SIGNR, pCAG-LSECtin, pCAG-3xSFTSVR, or pmcherry-C1. The electroporation conditions were 1200 V, 20-ms pulse width, and two pulses. Transfected cells were transferred to 2 mL of pre-warmed culture medium.

### 2.4 Protein extraction, immunoprecipitation, and western blotting

Cells were lysed in ice-cold RIPA lysis buffer (25 mM Tris-HCl [pH 7.4], 150 mM NaCl, 1% Non-idet P-40, 0.5% sodium deoxycholate, and 0.1% sodium dodecyl sulfate) containing a protease inhibitor mixture (GenDEPOT) and incubated for 1 h on ice. After centrifugation at 14,000 rpm for 10 min at 4°C, the protein concentration was determined using the BCA assay (Thermo Fisher Scientific Inc. #23222, #23224). Tissues (brain, heart, liver, kidney, spleen, lung, cervical lymph nodes, and thymus) of 6-8-week-old female wildtype (WT) and 3xTG mice were isolated from the mice, directly homogenized in 2x Laemmli sample buffer, and boiled for 10 min. For western blotting, the protein samples were separated by SDS-PAGE and transferred to a nitrocellulose membrane (Millipore). The membranes were blocked with 5% non-fat dry milk in TBS-T (Tris-buffered saline containing 0.1% Tween 20) for 1 h at 25°C and then probed with primary antibodies overnight at 4°C. After incubation with a horseradish peroxidase-conjugated secondary antibody, the membranes were developed using an ECL detection system. The membranes were probed with primary antibody against DC-SIGN (Abcam, ab218419, and Cell signaling,13193), DC-SIGNR (Abcam, ab169783, and ab232709), LSECtin (Santa Cruz, sc-65478, and Abcam, ab181196), Hsp90α/β (Santa Cruz, sc-13119), β-Actin (Santa Cruz, sc-47778), and GAPDH (Santa Cruz, sc-47724).

### 2.5 Immunocytochemistry (ICC)

Before seeding, a cover glass (Marienfield) was coated with a 1% (w/v) gelatin solution in PBS and incubated for 30 min at 37°C. After washing, the cells were fixed with 10% formalin in PBS for 15 min at room temperature (RT). After three washes with PBS, cells were permeabilized with 0.5% Triton X-100 in PBS for 10 min at RT. After three additional washes with 0.1% Triton X-100 in PBS, the cells were blocked with 5% BSA and 0.5% Triton X-100 in PBS for 30 min at RT. The cover glass was placed on parafilm inside a humid chamber and incubated overnight at 4°C with the following primary antibodies, which were diluted to 1:100 in 5% BSA and 0.5% Triton X-100 in PBS: DC-SIGN (Abcam, ab218419), DC-SIGNR (Abcam, ab169783), LSECtin (Santa Cruz, sc-65478). After washing the cells thrice with 0.1% Triton X-100 in PBS for 5 min per wash, they were incubated with secondary antibodies conjugated with fluorophores (Invitrogen, A32744 and A21207) diluted 1:100 in PBS with 0.1% Triton X-100. Following three additional washes with 0.1% Triton X-100 in PBS for 5 min each, the cells were mounted on glass slides using Fluoroshield with 4’,6-diamidino-2-phenylindole (DAPI) (Sigma-Aldrich, F6057). Conventional fluorescent imaging was performed using the Axio Observer Z1/7 (Carl Zeiss), and confocal images were captured using an LSM980 (Carl Zeiss).

### 2.6 *In vitro* infectivity assay

BJAB cells were infected with GFP-expressing SFTSV at a multiplicity of infection (MOI) of 1 and 48 h post-transfection. At 24 h post-infection, the cells were collected, washed with PBS, blocked with a homemade Fc blocker, and stained with specific antibodies in FACS buffer (PBS supplemented with 3% FBS). The antibodies used for staining were DC-SIGN (Abcam, Cambridge, UK; C209/1781), DCSIGNR (Abcam, Cambridge, UK; EPR11211), or LSECtin (Santa Cruz, Santa Cruz, CA, USA; SOTO-1). Secondary antibodies, Alexa594 conjugated anti-rabbit IgG or Alexa594 conjugated anti-mouse IgG (Invitrogen), were selected based on the host species of the primary antibodies. Primary and secondary antibodies were stained on ice for 20 and 30 min, respectively. Flow cytometry data were acquired using CytoFLEX S (Beckman Coulter) and analyzed using FlowJo software (BD). Live cells and singlets were gated for analyses using the Zombie Aqua Fixable Viability dye (BioLegend) and forward and side scattering characteristics.

### 2.7 Animal

Human SFTSVR transgenic (3xTG) mice were generated by the direct microinjection of a transgene into mouse embryos. All mice were maintained in heterozygous TG and homogenous WT conditions on a C57BL/6J background and housed in a specific pathogen-free animal facility at the Yonsei Laboratory Animal Research Center (YLARC). The mice were kept in a controlled environment with a 12-h light/dark cycle, fed a normal chow diet, and provided free access to food and water. The study protocol was approved by the Institutional Animal Care and Use Committee of Yonsei University (IACUC-202309-1732-01) and the Institutional Animal Care and Use Committee of Seoul National University (SNU-220512-6-1). The 3xTG mice were validated using PCR analysis of genomic DNA extracted from the tail tips of mice. The primers used for genotyping were as follows: DC-SIGN PCR forward, 5′-GGA TTC CGA CAG ACT CGA GGA-3′; and DC-SIGN PCR reverse, 5′-GAC TTA TGG AGC TGG GGA CCT-3′; DC-SIGNR PCR forward, 5′-GTA ACC GCT TCT CCT GGA TGG-3′; and DC-SIGNR PCR reverse, 5′-CAT CGA TTG TCG TTC CAG CCA-3′; LSECtin PCR forward, 5′-TTC TCT GTG CCA AAG ACG ACG -3′; and LSECtin PCR reverse, 5′-CTG AGA GAG ACT CCG TCC ACC -3′; Internal control PCR forward, 5′-GTA GGT GGA AAT TCT AGC ATC ATC C -3′; and internal control PCR reverse, 5′- CTA GGC CAC AGA ATT GAA AGA TCT -3′. The female 3xTg mice were divided into two age groups: young (≤ 20-week-old, *n* = 6) and aged (> 20-week-old, *n* = 5). Age-matched female WT mice (young *n* = 6, and aged *n* = 5) and Interferon α/β receptor (IFNAR) knockout (KO) mice (young *n* = 6, and aged *n* = 5) were used as controls. All mice were housed and maintained in a specific pathogen-free facility at Seoul National University College of Medicine.

### 2.8 RT-qPCR

Total RNA was extracted from tissue samples of 6–8-week-old female WT and 3xTG mice using the TRIzol reagent (Ambion) according to the manufacturer’s instructions. Complementary DNA (cDNA) was synthesized from 1 μg of total RNA using the RevertAid First Strand cDNA Synthesis Kit (Thermo Scientific, K1622), following the instructions of the manufacturer. The resulting cDNA was diluted 1:10 in nuclease-free water and used as the template for qPCR. qPCR was performed using a StepOnePlus Real-Time PCR System (Thermo Fisher Scientific) with SYBR Green PCR Master Mix (Bio-98020; Meridian Bioscience). The PCR reaction mixture consisted of 10 μL of 2x SYBR Green PCR Master Mix, 1 μL of each primer (10 μM), 1 μg of cDNA template, and nuclease-free water to a final volume of 20 μL. The relative gene expression was calculated using the delta-delta cycle threshold (ΔΔCt) method, with beta-actin as the reference gene. The primer sequences used for qPCR are listed in Supplementary Table 1. The qPCR protocol was repeated at least thrice to ensure reproducibility of the results. Statistical analyses were performed using GraphPad Prism (version 5.0.2), and data are presented as mean ± standard error of the mean (SEM). The significance threshold was set at *p* < 0.05. SFTV-infected mouse tissues were homogenized for 5 min using TRIzol reagent (Life Technologies). Mouse blood was centrifuged at 10,000 ×*g* for 10 min to separate serum, and then 250 μL of serum was added to 750 μL of Trizol LS reagent (Life Technologies). Total RNA was extracted according to the manufacturer’s instructions. RNA was reverse-transcribed into cDNA using the HiSenScript RH (-) RT Premix kit (Intron). The cDNA was quantified using the SensiFAST Probe Lo-ROX kit (Bioline) with primers (forward: 5′- CCTTCAGGTCATGACAGCTGG-3′, reverse: 5′-ACCAGGCTCTCAAT CACTCCTGT-3′) and detecting probe (5′-6FAM-AGCACATGTC CAAGTGGGAAGGCTCTG-BHQ1-3′) derived from the NP gene of SFTSV. qRT-PCR was performed on a Bio-Rad CFX connected real-time system (Bio-Rad).

### 2.9 Immunohistochemistry (IHC)

The spleen, kidney, and liver of 6–8-week-old female 3xTG and WT mice were fixed in 10% neutral-buffered formalin (Sigma-Aldrich, St. Louis USA, MO). After 24 h, the tissues were dehydrated thrice in a series of ethanol solutions of increasing concentrations up to 100% (70% ethanol for 40 min, 80% ethanol for 40 min, 95% ethanol for 40 min, and 100% ethanol for 1 h (thrice)). Following dehydration, the tissues were immersed in three different xylene immersions (for 1 h each time) followed by the infiltration in three different paraffin wax immersions (60°C, 1 h twice, and lastly 1 h 30 min) using the overnight program of the HistoCore PEARL automated tissue processor (Leica Biosystem). The tissues were embedded and sectioned at a thickness of 4 μm for immunostaining. For IHC, the sections were deparaffinized in different xylene immersions [5 min (twice)], rehydrated (100% ethanol for 2 min twice, 95% ethanol for 3 min, 70% ethanol for 2 min, 50% ethanol for 2 min, and distilled water for 4 min), and antigen-retrieved by heat (95°C) activation in 10 mM, pH 6.0 sodium citrate buffer for 10 min. Sections underwent IHC using standard protocols. An IHC Application Solutions Kit (Cell Signaling, #13079) was used for DC-SIGN (Cell signaling, 13193), DC-SIGN (Abcam, ab232709), and LSECtin (Abcam, ab181196) staining. Images were acquired using a Nikon Eclipse-80i microscope (Nikon, Tokyo, Japan) with an X40 (numerical aperture = 0.60) objective.

### 2.10 SFTSV challenge

The 10^5^ FFU of SFTSV in a 100 μL volume subcutaneously inoculated to young (*n* = 6) and aged (*n* = 5) female 3xTG mice, along with young (*n* = 6) and aged (*n* = 5) female WT C57BL/6J and IFNAR KO mice (*n* = 5). The weight loss and disease symptoms were monitored daily. Mice were sacrificed by cervical dislocation 3 days post-infection (DPI). Following sacrifice, various organs were harvested for quantitative RT-PCR and hematological analysis. Viral titers in the serum, spleen, and lymph nodes were quantified in young (*n* = 3) and aged (*n* = 4) 3xTG mice, young (*n* = 3) and aged (*n* = 4) WT mice, and IFNAR KO mice (*n* = 3) 3 days post-infection. Platelet counts were analyzed using a hematology analyzer BC-2800 (Mindray) in the same group at the same time points. All challenge experiments were performed at the Animal Biosafety Level 3 (ABSL-3) facility of the Department of Experimental Animal Research (DEAR), Seoul National University Hospital.

## 3 Results

### 3.1 Generation of the transgene construct co-expressing human DC-SIGN, DC-SIGNR, and LSECtin

**FIGURE 1:**
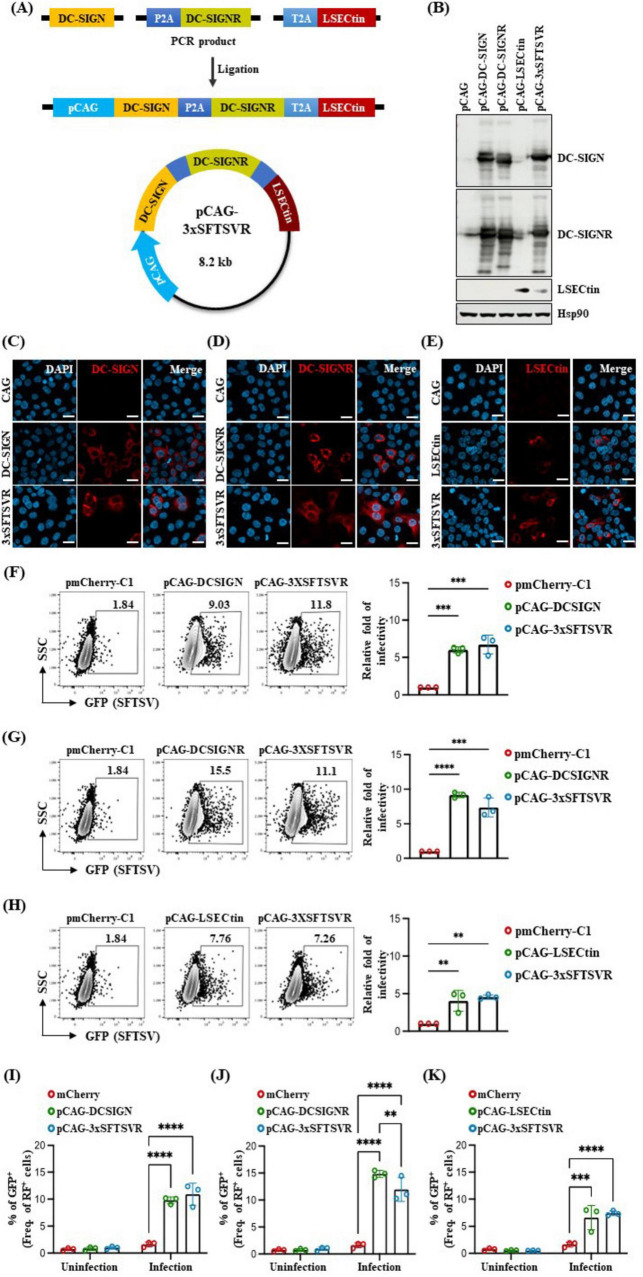
Generation of a transgene construct containing three SFTSV receptors and its expression. **(A)** Schematic illustration showing the strategy for generating the transgene construct of pCAG-hDC-SIGN-hDC-SIGNR-hLSECtin (pCAG-3xSFTSVR). pCAG, indicated by the bright blue arrow, contains a CMV enhancer element, chicken beta-actin promoter, and the splice acceptor of the rabbit beta-globin. Human DC-SIGN is colored yellow, human DC-SIGNR is green and human LSECtin is red. P2A is colored ocean blue, and T2A is colored sky blue. The tri-cistronic construct in a pCAG backbone with 2A peptides expresses human DC-SIGN, human DC-SIGNR, and human LSECtin. **(B–E)** Tri-cistronic construct expression test. Constructs were transfected into the HEK 293T cells, then the cells were collected 30 h after transfection for **(B)** western blot analysis and **(C–E)** fixed 24 h after transfection for immunocytochemistry. Nuclei were stained by DAPI. Scale bar = 20 μm. **(F–K)** Comparative analysis of SFTSV infectivity in transfected BJAB cells (MOI = 1, *n* = 3). Cells were transfected with various vectors: control pmCherry-C1 (**F–K**, Red), pCAG-hDCSIGN (**F,I**, green), pCAG-hDCSIGNR (**G,J**, green), pCAG-hLSECtin (**H,K**, green) or pCAG-3xSFTSVR (**F–K**, blue), and incubated for 48 h. Transfected cells were infected with GFP-expressing SFTSV and then analyzed by flow cytometry after 24 h post-infection. Cells were stained with anti-DCSIGN antibody **(F)**, anti-DCSIGNR antibody **(G)**, or anti-LSECtin antibody **(H)**. **P > 0.01, ***p < 0.001, ****p < 0.0001. A two-way ANOVA was performed to compare variables between groups.

To better understand the mechanism of SFTSV infection, we established a suitable SFTSV infection model in mice that expresses three human entry receptors: DC-SIGN, DC-SIGNR, and LSECtin. We engineered a construct (3xSFTSVR) by inserting cDNA fragments of these genes into the pCAGGS vector, controlled by a CMV enhancer and chicken beta-actin promoter (Figure 1A). To enhance the transcriptional efficacy of our target genes, we incorporated two 2A peptides derived from thosea asigna virus (T2A) and porcine teschovirus-1 (P2A) (Wang et al., 2015; Kim et al., 2011). The expression of the 3xSFTSVR construct was validated in western blot analysis, FACS analysis, and ICC after transfection of human 293T cells with the constructs (Figures 1B–K). Western blot analysis revealed that the high homology between DC-SIGN and DC-SIGNR resulted in detection by each respective antibody when only pCAG-DC-SIGN and pCAG-DC-SIGNR were expressed. However, the expression of pCAG-3xSFTSVR in 293T cells confirmed robust expression of DC-SIGN, DC-SIGNR, and LSECtin (Figure 1B). ICC further demonstrated effective expression of the 3xSFTSVR, comparable to individual receptor transfections (Figures 1C–E). We then assessed the impact of these receptors on SFTSV infectivity by transfecting BJAB cells with vectors encoding DC-SIGN, DC-SIGNR, LSECtin, 3xSFTSVR, or mCherry. FACS analysis confirmed that the 3xSFTSVR construct expressed all three receptor proteins as effectively as the individual constructs in BJAB cells (Figures 1F–H). Subsequently, the transfected cells were infected with GFP-expressing SFTSV at a multiplicity of infection (MOI) of 1 for 24 hours. Infection rates were determined by flow cytometry by comparing the proportion of SFTSV-infected (GFP-positive) cells within the transfected (red fluorescence-positive) population. Cells transfected with DC-SIGN or 3xTG exhibited approximately 6.02- and 6.73-fold increases in SFTSV infectivity, respectively, compared to control (Figures 1F, I). Furthermore, cells expressing DC-SIGNR demonstrated a 9.16- and 7.37-fold increase in infectivity, respectively (Figures 1G, J). While LSECtin-transfected cells showed a lower increase in infection rate than DC-SIGN and DC-SIGNR, both LSECtin and 3xSFTSVR transfected cells displayed a 4-fold increase (Figures 1H, K). Collectively, the expression of individual entry receptors alone was sufficient to enhance SFTSV infectivity, while cells expressing 3xSFTSVR exhibited the highest susceptibility to infection.

### 3.2 Establishment of an SFTSV receptors transgenic mouse model for *in vivo* study

**FIGURE 2:**
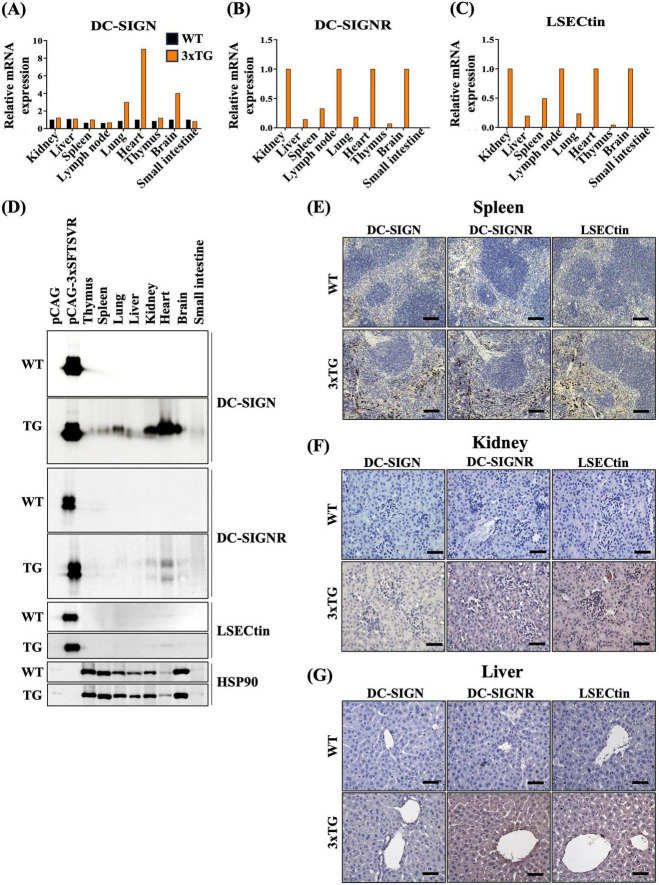
Expression of three SFTSV receptors in 3xTG mouse. **(A–C)** RT-PCR analysis demonstrated the relative mRNA levels of hDC-SIGN, hDC-SIGNR, and hLSECtin in the thymus, spleen, lung, liver, kidney, heart, brain, and small intestine of wildtype (WT, *n* = 1) and 3xTG mice (*n* = 1). **(D)** Western blot analysis revealed the expression of hDC-SIGN, hDC-SIGNR, and hLSECtin in the thymus, spleen, lung, liver, kidney, heart, brain, and small intestine of wildtype (WT, *n* = 1) and 3xTG (*n* = 1) mice. **(E–G)** Immunohistochemistry staining showed increased expression of the three human SFTSV receptors in the spleen, kidney, and liver of 3xTG mouse (*n* = 1) compared to WT mouse (*n* = 1). The scale bar for the spleen is 100 μm; for the kidney and liver, it is 50 μm.

To develop a mouse model expressing the three human SFTSV receptors (3xTG) to study SFTSV infectivity, we microinjected linearized DNA (3xSFTSVR) into pronuclei-stage mouse embryos. Founder 3xTG mice were bred with WT C57BL/6N (B6) mice to produce whole-body 3xTG offspring. Expression levels of DC-SIGN, DC-SIGNR, and LSECtin in both WT and 3xTG mice were evaluated using RT-PCR and Western blotting (Figures 2A–D). We observed significantly higher expression of these receptors in 3xTG mice compared to WT mice. To further validate the increased expression of the three receptors in 3xTG mice, we conducted immunohistochemical analyses on key organs, including the spleen, kidney, and liver (Figures 2E–G). The results confirmed a substantial increase in the expression of all three receptors across these organs in 3xTG mice compared to their WT counterparts. These 3xTG mice were subsequently used for further investigation of viral infections.

### 3.3 Susceptibility of 3xTG mice to SFTSV infection

**FIGURE 3:**
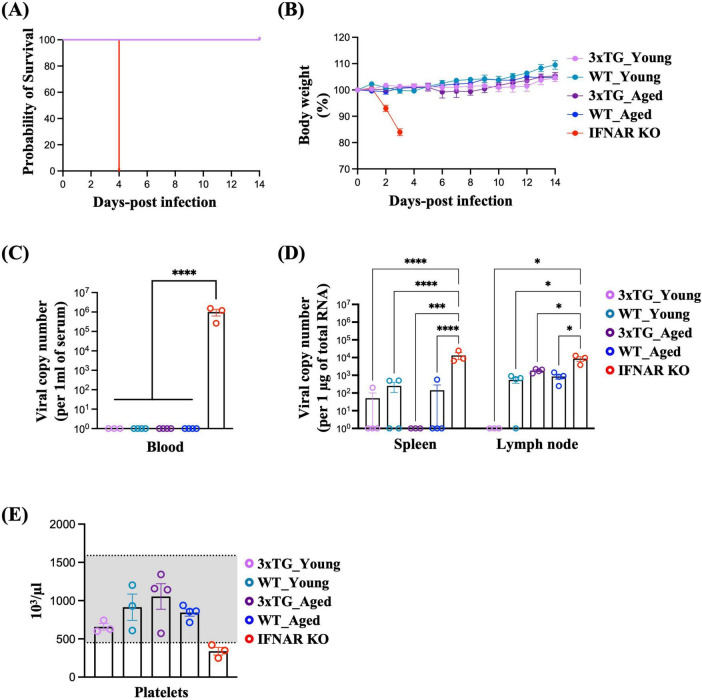
Low Susceptibility of 3xTG Mice to SFTSV Infection. Mice were subcutaneously infected with 10^5^ FFU of SFTSV. (A and B) Survival probability **(A)** and body weight **(B)** were monitored in young (*n* = 6) and aged (*n* = 5) 3xTG mice, along with young (*n* = 6) and aged (*n* = 5) WT C57BL/6J and IFNAR KO mice (*n* = 5), after SFTSV infection. **(C,D)** Viral titers in the serum **(C)**, spleen, and lymph nodes **(D)** were quantified in young (*n* = 3) and aged (*n* = 4) 3xTG mice, young (*n* = 3) and aged (*n* = 4) WT C56BL/6J mice, and IFNAR KO mice (*n* = 3) 3 days post-infection. **(E)** Platelet counts were measured in the same group at the same time points. *p < 0.05, ***P > 0.001, ****p < 0.0001. Two-way ANOVA was performed to compare the variables between groups [**(C,D)**].

To determine whether the expression of the three human SFTSV receptors (SFTSVRs) enhanced viral infectivity *in vivo*, we investigated the susceptibility of 3xTG mice to SFTSV infection. Age has been known to be a critical risk factor for SFTS, and the mortality rate increases dramatically with age (Ding et al., 2014). Thus, we prepared 3xTG mice into two age groups: young (≤ 20-week-old) and aged (> 20-week-old), along with age-matched wild-type (WT) controls. Each mouse was administered a subcutaneous injection of 10^5^ FFU of SFTSV. Over a 14-day observation period, we meticulously documented the changes in body weight. Contrary to results using a lethal model with IFNAR KO mice (Sun et al., 2021; Park et al., 2020), both WT and 3xTG mice exhibited 100% survival rates (Figure 3A). Moreover, neither WT nor 3xTG mice experienced weight loss and no differences were observed between the young and aged cohorts (Figure 3B). Three days post-infection, SFTSV was undetectable in the serum of both WT and 3xTG mice, except in IFNAR KO mice, which served as a positive control (Figure 3C). Concurrently, in some mice, the virus was present in the inguinal lymph nodes and spleen; however, the viral levels were comparable between WT and 3xTG mice and were significantly lower than those in IFNAR KO mice (Figure 3D). We further evaluated thrombocytopenia, a hallmark of SFTSV infection, by measuring platelet counts three days post-infection. Although IFNAR KO mice displayed platelet counts below the normal threshold, indicative of active infection, both WT and 3xTG mice maintained platelet counts within the normal range across all age groups (Figure 3E). In summary, the 3xTG mice displayed susceptibility to SFTSV infection equivalent to that of their WT counterparts, suggesting that the expression of the three human SFTSV receptors alone does not sufficiently enhance viral infectivity.

## 4 Discussion

The initial response to SFTSV infection engages the activation of both innate and adaptive immune systems, including robust changes in B cell and macrophage functionality (Suzuki et al., 2020; Luo et al., 2023). This compromised immune response can trigger a cytokine storm and severe inflammatory response syndrome (SIRS) (Luo et al., 2023; Sun et al., 2021; Bopp et al., 2020; Yang et al., 2022). Consequently, it can lead to disseminated intravascular coagulation and hemorrhage, multiple organ failure, and ultimately early mortality (Luo et al., 2023; Xu et al., 2021; Yang et al., 2022). Current treatments for SFTS are primarily focused on symptom management (Luo et al., 2023). Therapeutic interventions such as ribavirin, favipiravir, calcium channel blockers, immunoglobulin, glucocorticoid, and plasma exchange have shown limited response, benefiting only a subset of patients (Luo et al., 2023; Kim et al., 2016; Takayama-Ito and Saijo, 2020; Li et al., 2019; Yang et al., 2022; Shimojima et al., 2015; Lee et al., 2017; Nakamura et al., 2018). Despite these efforts, the lack of an approved vaccine against SFSTV remains a critical barrier to combating the disease effectively. To overcome this challenge, the development of robust animal models that accurately mimic the pathogenesis and immune response observed in human SFTS is of utmost importance. Such models will be essential in advancing SFTSV vaccine research and should be a high priority in future studies.

Recently, C-C motif chemokine receptor 2 (CCR2) was identified as the host entry receptor of SFTSV (Zhang et al., 2023). Zhang et al. (2023) demonstrated a strong correlation between SFTSV infectivity and CCR expression across multiple immune cell lines. Their work with CCR2 KO mice revealed a notably high survival rate post-SFTSV infection. However, despite the promising results, CCR2 KO mice or CCR2 antagonist-treated mice still showed early viral infection and associated body weight loss. Furthermore, these mice were pretreated with an anti-IFNAR1 antibody, highlighting their limitation in fully replicating the early antiviral responses observed in humans. These findings suggest that additional factors likely contribute to the complex mechanisms of SFTSV infection (Sun et al., 2021).

In our study investigating the susceptibility and progression of SFTSV in 3xTG mice, individual C-type lectin receptors —DC-SIGN, DC-SIGNR, and LSECtin —facilitated pathogen entry into both *in vivo* and *in vitro* systems. However, overexpression of the three receptors alone did not significantly enhance SFTSV infectivity compared to wild-type mice. Similarly, Park et al. (2023) developed a transient DC-SIGN-overexpressing mouse model using an adeno-associated virus delivery system, which demonstrated remarkably increased susceptibility to SFTSV. Despite its success in reproducing certain SFTS phenotypes, the numbers of platelets, red blood cells, and white blood cells in the DC-SIGN-overexpressing mice returned to base line by the sixth day post-infection (Park et al., 2023). Moreover, while human DC-SIGN expression in mouse bone marrow-derived dendritic cells enhances CD4^+^ T cell priming (Schetters et al., 2018), its expression in mycobacterial infection models was associated with reduced pathology (Schaefer et al., 2008). Interestingly, our findings align with observations from hepatitis C virus research, where the simultaneous overexpression of DC-SIGN and DC-SIGNR, serving as transmission receptors, failed to improve infection efficacy beyond that achieved with a single receptor (Lozach et al., 2004). Collectively, these results suggest that overexpressing multiple entry receptors does not guarantee enhanced infection efficacy. The inability to fully mimic human SFTS in our 3xTG mice likely stems from fundamental differences in innate immune responses between mice and humans (Zschaler et al., 2014; O’Shea and Visconti, 2000; Meyts and Casanova, 2021; Xu et al., 2021; Schetters et al., 2018; Schaefer et al., 2008; Park et al., 2023). To address these limitations, we propose incorporating human IFNAR1 expression in future models, given its crucial role in the pathogenesis and immune response to SFTSV infection (Wang et al., 2023; Li et al., 2024; Harari et al., 2014). This approach may provide a more accurate representation of human immune responses and improve the reliability of mouse models for studying SFTSV pathogenesis and therapeutic interventions.

## Data Availability

The datasets presented in this study can be found in online repositories. The names of the repository/repositories and accession number(s) can be found in this article/Supplementary material.

## Supplementary material

Supplementary Figure 1 Gating strategy of BJAB cells expressing three SFTSV receptors. Representative gating strategy for analyzing human DC-SIGN, DC-SIGNR, and LSECtin.
