# Supplementary material for: Limitations of a proper SFTSV mouse model using human C-type lectin receptors
Source: Front Microbiol. 2024 Dec 19;15:1452739. doi: 10.3389/fmicb.2024.1452739 (PMC11693710; doi:10.3389/fmicb.2024.1452739)
Supplement: Supplementary file 1 [file Table_1.docx]

**Limitations of a Proper SFTSV Mouse Model Using Human C-type Lectin Receptors**

**Table S1. List of the QPCR primers used in the study.**

| **Primers** | **Sequence（5’-3’）** |
| --- | --- |
| DC-SIGN-F | CCTCTGTTGCCCAGCTTCAA |
| DC-SIGN-R | TTTGTCGTCGTTCCAGCCAT |
| DC-SIGNR-F | TGTCTAACTCCCAGCGGAAC |
| DC-SIGNR-R | CCATCCAGGAGAAGCGGTTA |
| LSECtin-F | TTCGAGGGCTCCTGCTACTT |
| LSECtin -R | GCGTGTTCCGAGTGAGGAAG |
| Gapdh-F | TTCACCACCATGGAGAAGGC |
| Gapdh-R | CCCTTTTGGCTCCACCCT |
