# Supplementary figures and images for: Limitations of a proper SFTSV mouse model using human C-type lectin receptors
Source: Front Microbiol. 2024 Dec 19;15:1452739. doi: 10.3389/fmicb.2024.1452739 (PMC11693710; doi:10.3389/fmicb.2024.1452739)

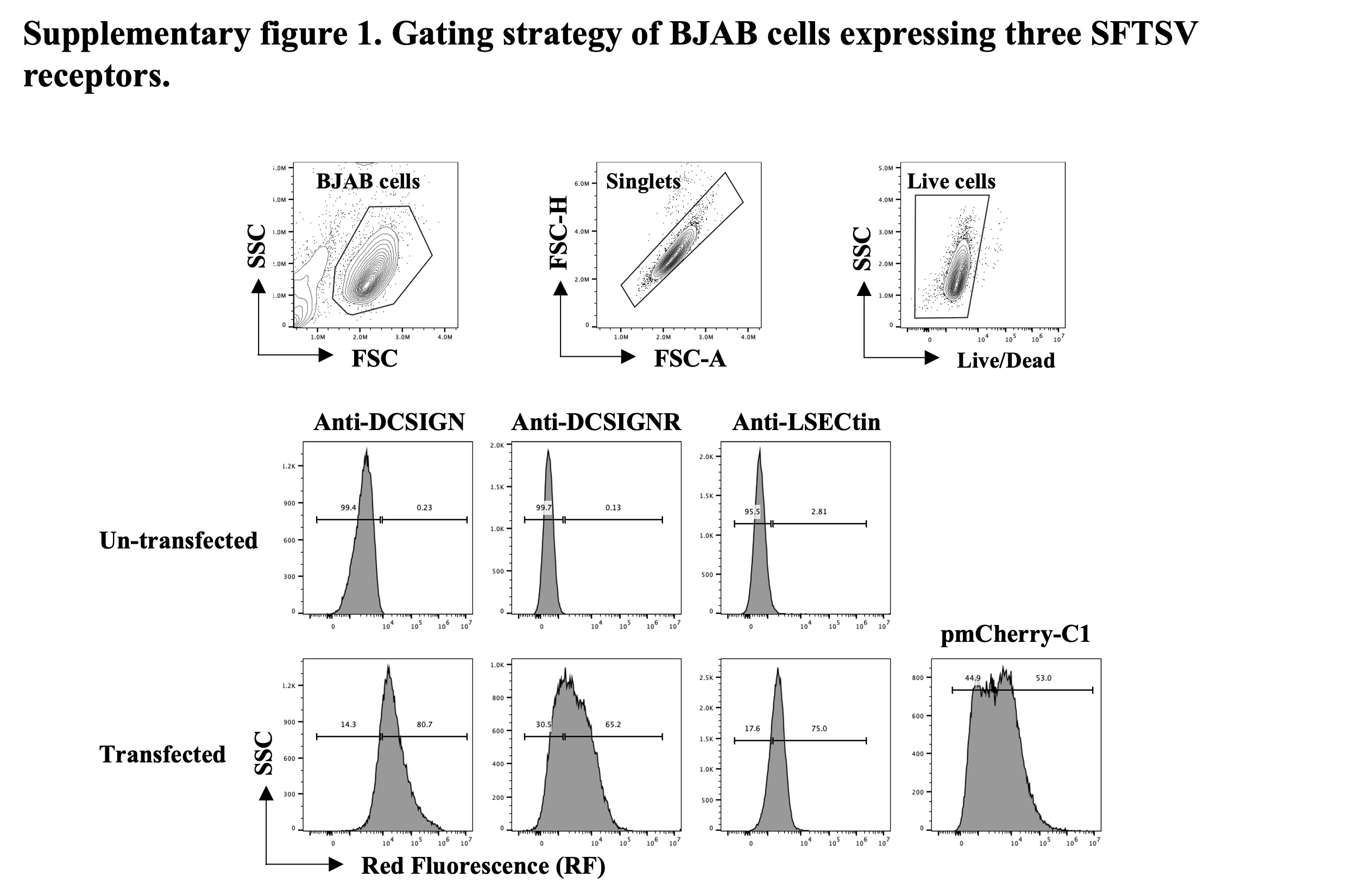

Supplement: Supplementary Figure 1 — Gating strategy of BJAB cells expressing three SFTSV receptors. Representative gating strategy for analyzing human DC-SIGN, DC-SIGNR, and LSECtin. [file Image_1.jpeg]
